# Usefulness of preoperative ice cream consumption and novel postoperative drainage management in patients undergoing left-sided neck dissection for thyroid cancer: a nonrandomized prospective study

**DOI:** 10.1007/s00595-023-02771-0

**Published:** 2023-12-05

**Authors:** Yusuke Mori, Hiroyuki Yamashita, Shinya Sato, Hisakazu Shindo, Seigo Tachibana, Takashi Fukuda, Misa Okamura, Atushi Yamaoka, Hiroshi Takahashi, Koichi Yoshimoto

**Affiliations:** 1 Department of Surgery, Yamashita Thyroid Hospital, 1-8 Simo-Gofukumachi, Hakata-Ku, Fukuoka City, Fukuoka 812-0034 Japan; 2 Department of Endocrinology, Yamashita Thyroid Hospital, Fukuoka City, Japan; 3 Department of Anesthesiology, Yamashita Thyroid Hospital, Fukuoka City, Japan

**Keywords:** Chyle leakage, Neck dissection, Thyroid cancer, Management

## Abstract

**Purpose:**

This study investigated the effects of ice cream consumption on chyle leakage after left lateral neck dissection in patients with thyroid cancer.

**Methods:**

A total of 491 patients with thyroid cancer underwent left lateral neck dissection with identification of the thoracic duct following ice cream consumption. Before closing the wound, the anesthesiologist increased the intrathoracic pressure to observe chyle leakage. If chyle leakage occurred postoperatively, the drain was removed using the drain negative pressure release test.

**Results:**

Postoperative chyle leakage was observed in 18 of the 491 patients who underwent left lateral neck dissection. We treated 17 patients conservatively and 1 patient surgically. Drains were removed within five days in all patients. After the drain negative pressure release test had been performed in eight patients, the drainage volume significantly decreased from an average of 175 ml to 31 ml per day. The average number of days until the removal of the drainage tube was 3.2 days. No perioperative complications were associated with ice cream consumption.

**Conclusions:**

In left lateral neck dissection for thyroid cancer, performing surgery following ice cream consumption does not completely prevent chyle leakage; however, early drain removal is possible because there is only mild leakage.

## Introduction

Chyle leakage or fistula after thyroid surgery has a lower incidence than complications such as recurrent laryngeal nerve palsy and hypoparathyroidism but may become a serious and occasionally fatal complication if not properly managed [1–3]. The management of chyle leakage includes conservative procedures and reoperations. Most chyle leakages or fistulas are conservatively managed by combining a low-fat diet or fasting, total parenteral nutrition, careful monitoring of fluids and electrolytes, chyle drainage, somatostatin analogs, and local pressure dressing [2, 3]. However, long-term hospitalization is generally required for recovery. Recently, clinical trials using radiological methods for patients in whom conservative treatments failed have shown favorable outcomes [4, 5]; however, these approaches have not been generally established as treatments.

Surgical intervention is required when conservative treatment is time-consuming or not curative [1, 2]. As it is challenging to understand the pathophysiology of individual patients with chyle leakage, surgeons cannot easily select the optimal treatment while considering the invasiveness, treatment period, and cost. Therefore, the treatment of chyle generally requires long-term hospitalization, enforcing restrictions on the patient's hospital stay and incurring high medical costs.

The lack of experience in individual facilities may also contribute to difficulty in establishing treatment methods. It is important to reduce chyle leakage occurrence using surgical techniques and treat such instances appropriately, based on chyle physiology, to avoid treatment-related difficulties. Visualization of the thoracic duct and some connecting lymphatic vessels can be improved after a fatty diet is consumed [6, 7]. We investigated the possibility of preventing chyle leakage using physiological properties to avoid injuring the lymphatic ducts during surgery, pinpoint small chyle leakage, and repair such cases by increasing intrathoracic pressure before wound closure [1]. If chyle leakage occurs postoperatively, the chyle may continue to flow out even while applying suction pressure to the drain. Thus, a drain negative pressure release (DNPR) test at the right time can reduce the duration of drain placement.

In 2017, we published a preliminary report in Japan on 177 cases involving ice cream consumption and have continued to accumulate more cases since then [8]. We herein report a method involving preoperative ice cream consumption to prevent chyle leakage in several patients with thyroid cancers and left lateral nodal metastases and discuss the management of patients with chyle leakage, focusing on the DNPR test.

## Methods

From June 2012 to May 2023, 935 patients with primary or recurrent thyroid cancer underwent therapeutic lateral neck dissection (LND). The 491 of these 935 who underwent left LND (LLND) following ice cream consumption were the primary subjects of this study. Of these, 383 patients underwent only left neck dissection, and 108 underwent bilateral neck dissection. The age of the 135 male and 356 female patients ranged from 10 to 86 years old (mean 51 years old) (Table 1).

**Table 1:** Characteristics of the 491 thyroid cancer patients who underwent left lateral neck dissection under ice cream preparation

Characteristics	Value (*n* = 491)
Gender	
Male	135
Female	356
Age (years)	
Median (range)	51 (10–86)
BMI	
Median (range)	23.1 (15.2–45.9)
Lateral neck dissection	
Left	383
Bilateral	108
Thyroid treatment	
Total thyroidectomy (Including residual thyroidectomy)	439
Pathology	
Papillary carcinoma	468
Follicular carcinoma	7
Medullary carcinoma	9
Poorly differentiated carcinoma	2
Undifferentiated carcinoma	5
Duration of postoperative admission (days)	5.1
Duration of deviation from clinical path (days)	+ 0.1

*BMI* body mass index

The study protocol was approved by the Ethics Committee of Yamashita Thyroid Hospital. All patients provided their written informed consent to participate in the study.

**Fig. 1:**
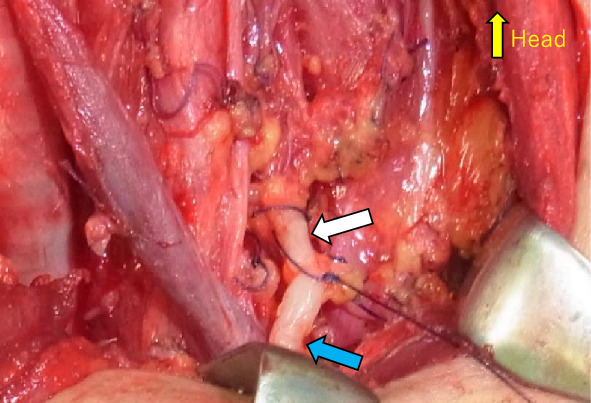
A white and dilated thoracic duct during left lateral neck dissection. The white arrow indicates the central side of the duct, and the blue arrow indicates the peripheral side

Patients undergoing LLND were provided ice cream containing long-chain fatty acids (commercially available ice cream intake: volume, 90 ml; fat content, 11.8%) 3 h before surgery to prevent chyle leakage or fistula [7]. After ingestion of ice cream, the thoracic duct was confirmed to be an opaque, translucent vessel (Fig. 1). Meticulous dissection was performed in region IV while identifying the thoracic and lymphatic ducts. When processing the lymphatic vessels, 4–0 Vicryl^®^ was used for ligature and suturing, and Harmonic^®^ (ETHICON Endo-Surgery, Inc., Cincinnati, OH, USA) or LigaSure^®^ (Medtronic Inc., Dublin, Ireland) was used for sealing and incision. Before closing the wound, an anesthesiologist increased the airway and intrathoracic pressure to 30–40 mmH_2_O to check for chyle leakage or enable repair. A 3.5-mm Sumitomo Bakelite drain (Sumitomo Bakelite Co., Ltd., Tokyo, Japan) was used for drainage.

**Fig. 2:**
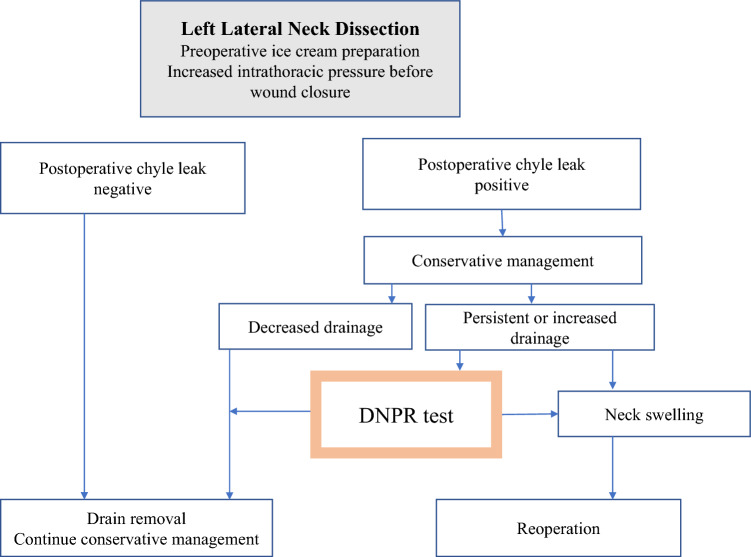
Our protocol for postoperative chyle leakage after left lateral neck dissection

Figure 2 shows the treatment policy after LLND. Chyle leakage was diagnosed based on the appearance of milky-white turbidity in the drain content, accompanied by an increase in volume after meal initiation. The diagnosis was confirmed by several surgeons.

The DNPR test was attempted if the drainage volume was either unchanged or increased without neck swelling due to chyle with or without local pressure dressing. The drain was removed once the volume decreased during the DNPR test.

### Statistical analyses

Wilcoxon’s signed-rank test was used to compare drain volumes before and after the DNPR test. All statistical analyses were performed using the JMP software program (version 17.0; SAS Institute Inc., Cary, NC, USA). Differences were considered statistically significant when the *P*-value was < 0.05.

## Results

Postoperative chyle leakage was observed in 21 of the 935 (2.2%) total patients and 18 of the 491 (3.7%) patients who underwent LLND following ice cream consumption. Of the 21 affected patients, 3 had right-sided and 18 left-sided chyle leakage (Table 2). Given that the amount of chyle in the 3 patients with right-sided leakage was small (average daily volume, 34 ml), allowing the drain to be removed within 3 days, and the primary focus of this study was the use of preoperative ice cream to prevent chyle leakage, we analyzed the data of the 18 patients with left-sided chyle leakage.

**Table 2:** Characteristics of the 18 patients with chyle leakage

Characteristics	Value
Gender	
Male	1
Female	17
Age (years)	
Median (range)	44 (19–75)
BMI	
Median (range)	20.5 (15.9–30.1)
Surgery	
Primary	15
Recurrent	3
Lateral neck dissection	
Left	12
Bilateral	6
Thyroid treatment	
Total thyroidectomy	16
Left IV lymph node metastasis (none/single/multiple)	2/8/8
Extra-lymphatic invasion of IV lymph nodes	5
Intraoperative lymphatic duct visibility	18
Maximum drainage volume per day (ml/day) (median)	50–630 (80)
Time until drain removal	3.2
Treatment	
Conservative	17
Reoperation	1
Post-operative admission (days)	5.4
Days of deviation from clinical path	+ 0.4
Fat-restricted diet	14
Fasting	0
Local pressure dressing	0
Special Treatment (somatostatin analogs, lymphangiography)	0

*BMI* body mass index

The main characteristics of the 491 patients are summarized in Table 1. Of the 18 evaluated patients with chyle leakage, 17 were treated conservatively, and 1 required reoperation (Table 3). Intraoperative chyle leakage was observed in 1 of the 18 cases and was managed with ligation. This patient showed improvement with conservative postoperative treatment.

**Table 3:** Summary of the characteristics of the 18 patients

								Management						
								DNPR test						
No	Age (years) /Sex	BMI	Dissection method	Total thyroidectomy	Date of chyle diagnosis (POD)	Maximum daily drainage volume(ml)	Date of drain removal (POD)	DNPRt	DNPRt start (POD)	Period from DNPRt to drain removal (day)	Pressure dressing	nutritional modification	Surgical intervention	Hospital length of stay(day)
1	66/F	22.1	LND(L),CND	Yes	1	50	2	No	–	–	Yes	–	No	5
2	30/F	19.0	LND(L),CND	Yes	2	640	5	Yes	3	2	Yes	MCT	No	8
3	52/F	23.2	LND(L),CND	Yes	1	70	3	No	–	–	Yes	–	No	6
4	60/F	20.2	LND(L),CND	Yes	1	70	3	No	–	–	Yes	–	No	5
5	44/F	30.1	LND(B),CND	Yes	1	400	-	No	–	–	Yes	MCT	Yes, day1	7
6	32/F	17.8	LND(B),CND	Yes	1	90	3	Yes	2	1	Yes	MCT	No	5
7	37/F	19.3	LND(L), CND	Yes	1	60	2	No	–	–	Yes	–	No	5
8	19/F	15.9	LND(L)	No	1	360	3	Yes	2	1	Yes	MCT	No	5
9	43/F	19.2	LND(L)	No	1	120	4	Yes	3	1	Yes	MCT	No	6
10	57/F	23.6	LND(L), CND	Yes	1	80	3	Yes	2	1	Yes	MCT	No	5
11	62/F	21.1	LND(B), CND	Yes	1	80	5	No	–	–	Yes	MCT	No	6
12	73/M	23.7	LND(B), CND	Yes	1	190	3	Yes	2	1	Yes	MCT	No	6
13	31/F	20.1	LND(L), CND	Yes	1	60	3	No	–	–	Yes	MCT	No	5
14	27/F	23.8	LND(B), CND	Yes	1	150	5	Yes	2	3	Yes	MCT	No	5
15	35/F	21.3	LND(L), CND	Yes	1	60	3	No	–	–	Yes	MCT	No	5
16	62/F	20.6	LND(L), CND	Yes	1	40	3	NO	–	1	Yes	MCT	No	5
17	75/F	20.3	LND(B), CND	Yes	1	100	3	No	–	1	Yes	MCT	No	5
18	28/F	19.8	LND(L)	Yes	1	100	3	Yes	2	1	Yes	MCT	No	5

*M* male, *F* female, *BMI* body mass index, *LND* lateral neck dissection, *CND* central neck dissection, *L* left, *B* bilateral, *POD* postoperative day, *DNPRt* drain negative pressure release test, *MCT* medium-chain triglyceride (low-fat diet)

**Fig. 3:**
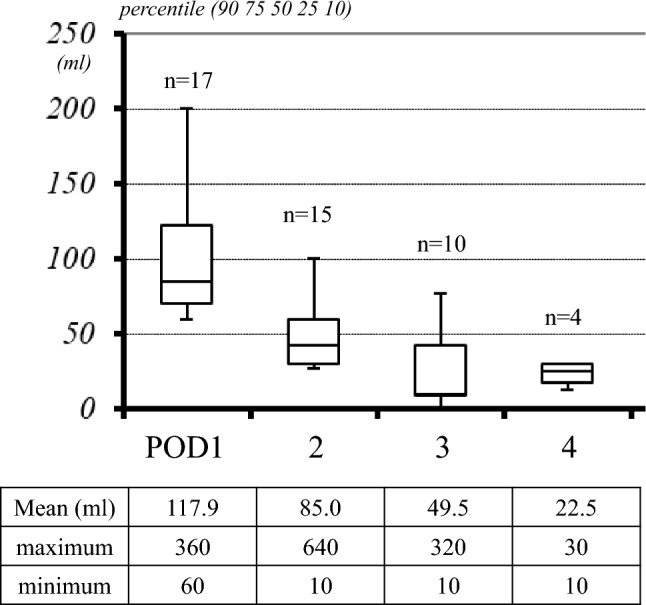
Changes in the daily drainage volume (ml) in 17 patients with chyle leakage

**Fig. 4:**
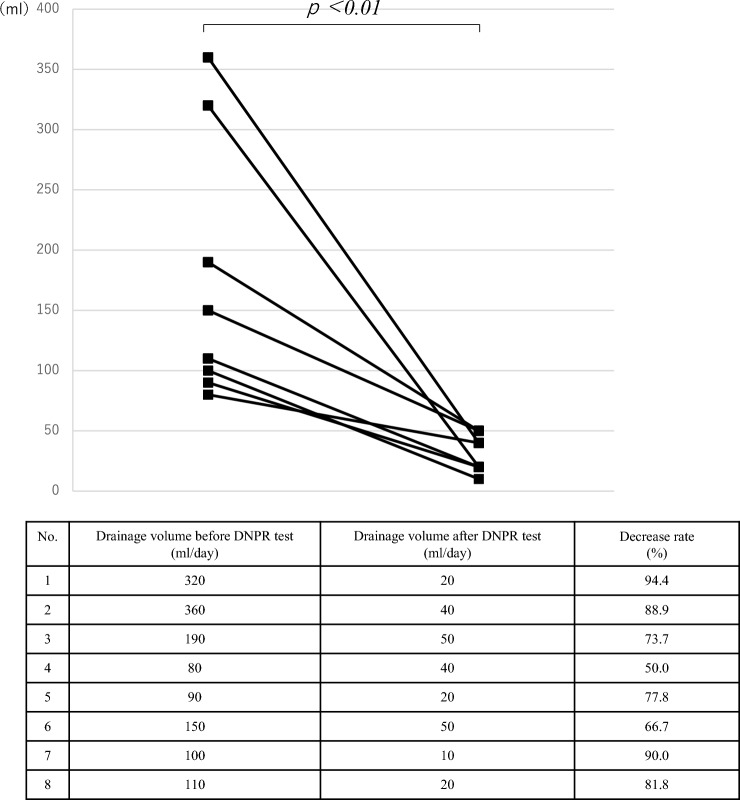
Drainage volume and its decreased rate before and after the drain negative pressure release test

Figure 3 shows the changes in drainage volume in these 17 patients. Data on postoperative day (POD) 0 were excluded because chyle appeared after meals on the first day of surgery. The average daily drainage volumes of the 17 patients were 118, 85, 50, and 23 ml on the first, second, third, and last days, respectively. The drain was removed on POD 5 in all 17 patients (Fig. 3). DNPR testing in 8 patients with persistent or increased drainage volumes revealed that the drainage volume significantly decreased from an average of 175 ml to 31 ml per day (*P* < 0.01, Fig. 4). None of the patients showed chyle leakage or recurrence of neck swelling after drain removal. None of the patients had fasted. The average time required to remove the drain was 3.2 days. The mean postoperative hospital stay of the 17 patients was 5.4 days.

In the patient who underwent reoperation, 400 ml of chyle discharge was observed 12 h after the first operation, accompanied by neck swelling. We did not expect an effect of local pressure dressing because her body mass index was relatively high at 30.1 kg/m^2^, and we decided to perform a reoperation on POD 1 following ice cream consumption. Because we found chyle leakage from the thoracic duct, which should have been ligated during the initial surgery, double ligations were performed on the leaking vessel. The drain was removed on POD 2, and the patient was discharged from the hospital on POD 5 after the initial surgery.

## Discussion

Chyle leakage or fistula is a troublesome complication usually associated with LLND for thyroid cancer [9]. Several measures have been studied for intraoperative lymphatic treatment in neck dissection, especially in the area around the venous angle, where the main trunk of the lymph merges [10]. For chyle leakage prevention, ligation of lymph vessels, including the surrounding tissue, is generally accepted. Monden et al. [11] reported that chyle leakage was safely treated with an energy device such as Harmonic Focus® (ETHICON Endo-Surgery, Inc.). However, Wang et al. [12] stated that lymphatic vessel treatment with energy devices is insufficient to prevent chyle leakage. Furthermore, several studies reported chylothorax following endoscopic lung or esophageal cancer resection using certain energy devices; therefore, such devices are not completely reliable [13, 14]. Zhang et al. [15] recently reported the usefulness of pedicled omohyoid flap coverings in preventing postoperative lymph node or chyle leakage. However, their study enrolled only a few patients, and the procedure was complicated. Thus, the generalizability of the findings is questionable [15]. As mentioned above, no effective chyle prevention method has yet been established.

The primary reason for the difficulty in preventing chyle leakage is the anatomy of lymphatic vessels and their complex networks [1, 16, 17]. Lymphatic vessels with a diameter of ≥ 0.2 mm are composed of smooth muscle and fibrous tissues, whereas thin lymphatic vessels have insufficient smooth muscle and fibrous tissues and form fragile tubular organs [18]. Prolonged fasting before surgery causes lymphatic vessel collapse, decreased lymphatic flow, and colorless and transparent lymphatic fluid. An effective way to avoid chyle leakage is to identify and ligate lymphatic ducts; however, it is difficult to distinguish lymphatic vessels from the surrounding connective tissues following prolonged fasting.

After experiencing complex cases of chyle leakage, we began providing ice cream preoperatively to prevent chyle leakage or fistula in patients scheduled to undergo LLND. Ice cream containing long-chain fatty acids is chylomicronized in the small intestine and increases lymphatic flow 3- to tenfold [6, 18]. In the study by Shackcloth et al. [7], patients were provided ice cream three hours before surgery; this safe method does not require special equipment and identifies the thoracic duct. This approach is especially indicated in patients with severe lymph node metastasis in region IV and those with postoperative chyle.

The frequency of chyle leakage varies according to the state of lateral neck lymph node metastasis and the extent of surgery. In this study, LND was performed only in patients with clinical lymph node metastasis to the lateral neck, and most patients underwent total thyroidectomy. The incidence rate of postoperative chyle leakage in this study was 2.2%, which is not superior to the rates reported in previous studies (2.9–8.3%) [12, 19–22]. However, in our patients, the degree of chyle leakage was mild, and most did not require reoperation or long-term hospitalization. The mean time to drain removal was 3.2 days, which is approximately 1 day longer than the 2 days specified in our postoperative path. Furthermore, there was no fasting, long local pressure dressing, or use of expensive medications. We want to emphasize that our prospective study is based on a novel treatment strategy aimed at preventing chyle leakage and managing its occurrence, and the number of patients in this study was higher than in other retrospective studies. According to previous reports, conservative treatment was performed in 50–89%, and reoperation was performed in 11–50% of patients [12, 19–22]. In addition, the median time to drain removal after conservative management was 21.5 (5–60) days, 11.3 days, and 12 days (range 8–22 days) in studies by Saito et al. [20], Roh et al. [21], and Glenn et al. [22], respectively. Our results may be explained by the small size of the leaking lymphatic vessels and the implementation of the DNPR test.

Prior to the present study, we performed the DNPR test and achieved successful outcomes in our patients, including a consultation case from another facility. In the present study, several patients had only a small amount of chyle, as ice cream was consumed before the surgical procedures. Although the DNPR test seemed irrelevant in the present patients, the amount of chyle was significantly reduced after the test in patients with persistent or increased chyle. The physiological internal pressures are 0–15 mmHg for large lymphatic vessels, such as the thoracic duct, and 0–0.5 mmHg for other lymphatic vessels [23]. Under these circumstances, drain suction pressure increases the wound pressure, which causes persistent chyle leakage. It is presumed that once the wound pressure is higher than the intralymphatic pressure, owing to the release or clamping of the drain, the amount of chyle is significantly reduced, thus allowing for early drain removal. We believe that such hypotensive lymphatics are easily healed by wound pressure. Conversely, persistent negative pressure in the drain is expected to form an iatrogenic fistula between the lymphatic vessels and wound. The DNPR test is helpful in patients with chyle leakage or fistulas from the hypotensive lymphatic vessels. In our patients, the timing of starting the DNPR test was left to the discretion of the attending surgeons; however, our results suggest that DNPR testing should have been performed considerably earlier than it actually was in our study. It may be possible to stop drain suction when diagnosing mild chyle in patients undergoing LLND after ice cream consumption. If it does, chyle leakage should not be considered a serious complication.

One patient required reoperation for chyle leakage from the thoracic duct, which was ligated during the initial surgery. The following mechanisms may trigger the loosening of the thoracic duct ligation. The thoracic duct is highly elastic and prone to dilation and collapse. Ice cream consumption enhanced the thoracic duct, as confirmed intraoperatively; however, postoperative fasting severely shrank and loosened the ligature. Finally, chyle leakage occurred in the ruptured thoracic duct after the oral intake. Considering the possibility of lymphatic vessel collapse during postoperative fasting, it is better to perform double ligature of dilated lymphatic vessels in cases with ice cream consumption. Early determination of the effectiveness of conservative treatment is critical to avoid prolonged hospitalization and special treatment for patients with chyle leakage. There may be pros and cons regarding the timing until reoperation, but considering the restrictions on the patient's hospital stay, we believe it is better to perform it earlier than it is generally performed. If the reason for delaying reoperation is chyle curability, we recommend LLND be performed following ice cream consumption to familiarize surgeons with the anatomy of the thoracic duct. However, based on our experience, ice cream consumption likely reduces the number of patients who will require reoperation for chyle leakage.

Some studies have reported the efficacy of using somatostatin analogs for postoperative chyle [24, 25]. However, in Japan, the use of these drugs for managing chyle is not covered by national health insurance and requires ethics committee approval. Furthermore, these drugs are expensive and place a financial burden on patients, even if they can be used. We recommend the DNPR test be performed in patients who are hesitant to undergo reoperation before using somatostatin analogs.

One limitation of our study is its nonrandomized design, as we initially thought that chyle leakage could be prevented in almost all patients following ice cream consumption. Therefore, a randomized study design was not considered appropriate. We believe that groundbreaking treatment can be established in various cases by concentrating on ice cream consumption. Furthermore, our proposed techniques have potential applications in treating other cervical, thoracic, and abdominal surgeries and are not limited to thyroid surgery.

Our large prospective study concluded that ice cream consumption before LLND did not completely prevent chyle leakage in thyroid cancer patients. However, the degree of chyle leakage was mild owing to the avoidance of damage to major lymph vessels, and the drain was able to be removed within a short time with minimal burden on the patients. We recommend this simple and safe preoperative procedure for patients with malignant tumors requiring neck dissection, especially those with clinical lymph node metastases in region IV and those scheduled for reoperation for chyle leakage or fistula. Timely DNPR testing is also recommended to reduce the duration of drain placement.

## Data Availability

The datasets generated and/or analyzed during the current study are available from the corresponding author upon reasonable request.
